# Association of Postoperative Neutrophil Lymphocyte Ratio (NLR) and Monocyte Lymphocyte Ratio (MLR) with the Presence of Osteoporosis in Japanese Patients after Hip Fracture Surgery: A Retrospective Cohort Study

**DOI:** 10.1155/2021/5524069

**Published:** 2021-09-15

**Authors:** Hirofumi Bekki, Takeshi Arizono, Daiki Hama, Akihiko Inokuchi, Takahiro Hamada, Ryuta Imamura

**Affiliations:** ^1^Department of Orthopaedic Surgery, Kyushu Central Hospital of the Mutual Aid Association of Public School Teachers, Fukuoka, Japan; ^2^Department of Orthopaedic Surgery, National Kyushu Medical Center, Fukuoka, Japan

## Abstract

**Background:**

The diagnosis of osteoporosis is based on bone mineral density measurements expressed as a percentage of the young adult mean (YAM) in Japan. Osteoporosis is defined as YAM <70%, and intervention is recommended at this cutoff. Because osteoporosis has a strong association with systemic metabolic disorders, we postulated that patients with YAM <70% had higher inflammatory biomarker concentrations owing to the higher systemic stress compared with YAM >70%.

**Method:**

We retrospectively reviewed 94 patients with low-trauma hip fractures. Blood examinations were performed on postoperative day (POD) 1 and POD 7. We used neutrophil lymphocyte ratio (NLR) and monocyte lymphocyte ratio (MLR) to evaluate postoperative recovery. After dividing the 94 patients into two groups according to a YAM cutoff of 70%, we compared the differences in NLR and MLR.

**Results:**

On POD 1, patients with YAM >70% had a median NLR of 5.7 and a median MLR of 0.66, which were significantly lower than for patients with YAM <70% (8.8 and 0.9, respectively). Similarly, on POD 7, patients with YAM >70% had a median NLR of 2.0 and a median MLR of 0.31, which were significantly lower than for patients with YAM <70% (3.5 and 0.43, respectively).

**Conclusion:**

A YAM cutoff of 70% is an appropriate intervention threshold regarding postoperative recovery after hip fracture surgery. *Mini-Abstract*. Patients with YAM >70% showed lower NLR and MLR on POD 1 and POD 7. A YAM cuffoff of 70% is an appropriate intervention threshold regarding postoperative recovery after hip fracture surgery.

## 1. Introduction

In Japan, the criteria for diagnosing osteoporosis prepared by the Japanese Society for Bone and Mineral Research are based on bone mineral density (BMD) measurements expressed as a percentage of the young adult mean (YAM) for women [1]. Osteoporosis is defined as <70% of the young adult mean percentage (%YAM) for the mean BMD in healthy young people for the lumbar spine or femoral neck [2]. Intervention is recommended at a YAM of 70% (*T*-score: −2.7 standard deviation) at the femoral neck [3].

Because hip fractures are associated with high morbidity and mortality, patients require emergency surgical care [4]. Considering that osteoporosis is related to systemic metabolic disorders, such as hypertension, diabetes, and pulmonary and renal dysfunction [5–8], patients with osteoporosis may undergo serious stress after surgery owing to the operative invasiveness. The purpose of this study was to confirm the hypothesis that a YAM cutoff of 70% is appropriate regarding postoperative recovery for hip fractures.

## 2. Materials and Methods

### 2.1. Patients

In this retrospective cohort study, we reviewed 94 patients with low-trauma hip fractures (45 with trochanteric fractures, 49 with femoral neck fractures) treated at a single medical institution in Japan for 9 months from April 2020. We performed bipolar hip arthroplasty (BHA) or open reduction and internal fixation (ORIF) for hip fractures and ORIF for trochanteric fractures. YAM in the nonfractured femoral neck was measured by using the Horizon DXA System bone densitometer (Hologic Inc., Marlborough, MA). This was because the femoral neck is a consistently significant predictor of hip fractures, and the discriminant power was better than that measured at the lumbar spine [9].

Patients comprised 76 women and 18 men with a median age of 88 years (range, 61–105 years). The median YAM was 54% (47%–67%); 77 patients had a YAM <70%, and 17 patients had a YAM >70%. The median body mass index (BMI) was 19.8 kg/m^2^ (range, 12.5–30 kg/m^2^). In accordance with the American Society of Anesthesiologists Physical Status (ASA-PS) scale, 51 cases were classified as grade II and 43 cases were classified as grade III. Chronic diseases, namely, diabetes mellitus (DM) and hypertension (HT), were also evaluated; 14 patients had DM, and 59 had HT. This retrospective study was approved by the Kyushu Central Hospital review board (approval number: 21–1).

### 2.2. Laboratory Data Analysis

Blood examinations were performed on postoperative day (POD) 1 and 7. We used the neutrophil lymphocyte ratio (NLR) and monocyte lymphocyte ratio (MLR) to evaluate postoperative recovery. The association of postoperative NLR and MLR were clarified with the presence of osteoporosis. First, to confirm our hypothesis, we divided the 94 patients into two groups according to a YAM cutoff of 70%. In addition, we divided 77 patients with a YAM <70% into two groups according to a YAM cutoff of 50% (the median value among the 77 patients) and compared NLR and MLR. We then created a receiver operating characteristic (ROC) curve to predict which laboratory data were strongly correlated with YAM.

### 2.3. Statistical Analysis

All data were expressed as median and 25%–75% interquartile range (IQR). Differences between groups were evaluated using Fisher's exact test or Pearson's chi-square test. Nonnormally distributed variables were evaluated using the independent Wilcoxon signed-rank test. A *p* value of <0.05 was considered statistically significant. Data analyses were performed using the JMP statistical software package (ver. 15; SAS Institute, Cary, NC).

## 3. Results

The patients' demographic data are summarized in Table 1. The BMI in patients with YAM >70% was significantly higher than that in the YAM <70% group (21.9 vs. 19.5, respectively; *p*=0.006). There were no significant differences in any of the other clinical parameters between the two groups, namely, sex, age, ASA-PS, type of fracture, surgical procedures, and medical history.

**Figure 1:**
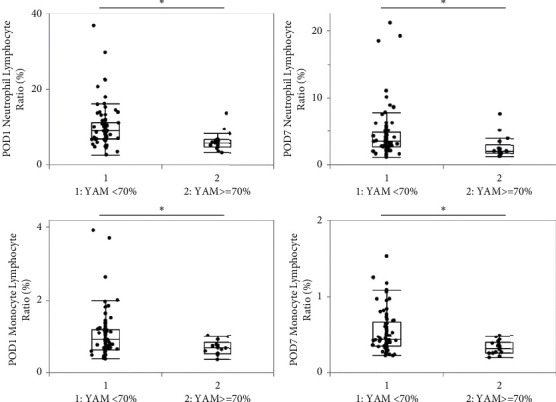
Comparison of neutrophil lymphocyte ratio (NLR) and monocyte lymphocyte ratio (MLR) according to a young adult mean (YAM) cutoff of 70%. Patients with YAM >70% had lower NLR and MLR values on postoperative day (POD) 1 and POD 7. ^*∗*^*P* < 0.01.

The comparison of NLR and MLR according to a YAM cutoff of 70% is shown in Figure 1. On POD 1, patients with YAM >70% had a median NLR of 5.7 (4.7–6.4) and a median MLR of 0.66 (0.51–0.82), which were significantly lower than for patients with YAM <70% (5.7 vs. 8.8, *p* < 0.001 and 0.66 vs. 0.9, *p*=0.0075, respectively). Similarly, on POD 7, patients with YAM >70% had a median NLR of 2.0 (1.7–2.9) and a median MLR of 0.31 (0.25–0.39), which were significantly lower than for patients with YAM <70% (2.0 vs 3.5, *p*=0.001 and 0.31 vs 0.43, *p*=0.0005, respectively).

**Figure 2:**
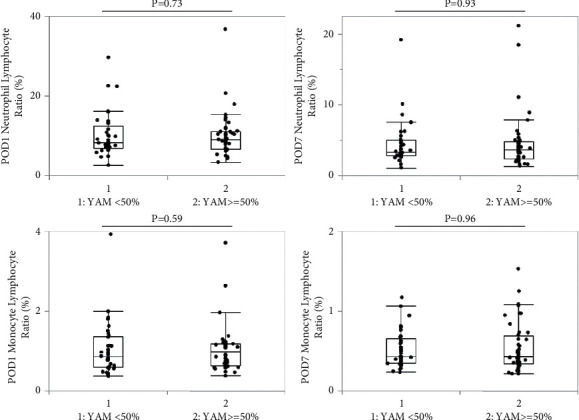
Comparison of neutrophil lymphocyte ratio (NLR) and monocyte lymphocyte ratio (MLR) according to a young adult mean (YAM) cutoff of 50%. On postoperative day (POD) 1 and POD 7, there was no difference for both NLR and MLR between patients with YAM >50% or YAM <50%.

The comparison of NLR and MLR according to a YAM cutoff of 50% is shown in Figure 2. On POD 1, patients with YAM <50% had a median NLR of 8.1 (6.8–12.4) and MLR of 0.86 (0.58–1.37). Patients with YAM ranging from 50% to 70% had a median NLR of 9.1 (6.7–11.0) and a median MLR of 0.99 (0.63–1.18). There was no significant difference in both NLR and MLR between the two groups. Similarly, the data for POD 7 showed no difference between the two groups (NLR: 3.3 vs. 3.6 and LMR: 0.42 vs. 0.43, respectively).

**Figure 3:**
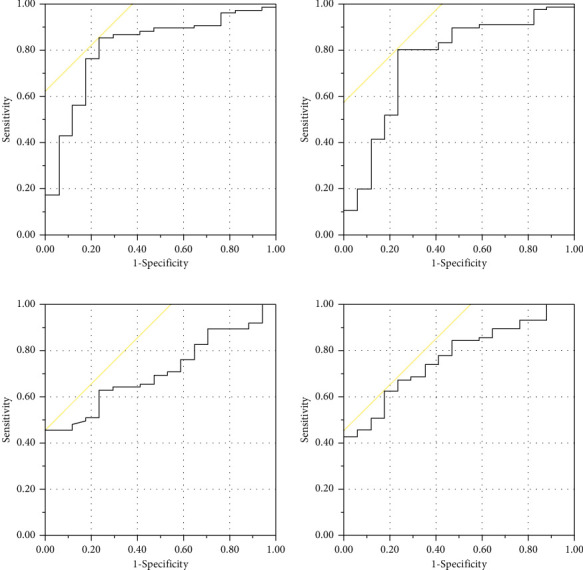
Receiver operating characteristic curves for neutrophil lymphocyte ratio (NLR) and monocyte lymphocyte ratio (MLR). (a) NLR of POD1. Sensitivity, 0.85; 1−specificity, 0.24; and area under the curve, 0.81. (b) NLR of POD7. Sensitivity, 0.81; 1−specificity, 0.24; and area under the curve, 0.76. (c) MLR of POD1. Sensitivity, 0.45; 1−specificity, 0.00; and area under the curve, 0.71. (d) MLR of POD7. Sensitivity, 0.62; 1−specificity, 0.18; and area under the curve, 0.77.

The results of the ROC curve analysis are shown in Figure 3. For NLR, data from POD 1 and POD 7 demonstrated that the values, 6.31 and 2.51, respectively, were predictive factors for YAM <70% (sensitivity, 0.85; 1−specificity, 0.24; area under the curve, 0.81 vs. sensitivity, 0.81; 1−specificity, 0.24; and area under the curve, 0.76). For MLR, data from POD 1 and POD 7 demonstrated that the values, 1.04 and 0.41, respectively, were predictive factors for YAM <70% (sensitivity, 0.45; 1−specificity, 0.00; area under the curve, 0.71 vs. sensitivity, 0.62; 1−specificity, 0.18; and area under the curve, 0.77).

## 4. Discussion

In this retrospective cohort study, we confirmed the hypothesis that a YAM cutoff of <70% was appropriate regarding postoperative recovery in Japanese patients after hip fracture surgery. We used NLR and MLR as biomarkers for postoperative invasiveness. Patients with YAM >70% had lower NLR and MLR values on both POD 1 and POD 7, suggesting that a YAM of 70% was an appropriate intervention threshold regarding postoperative recovery.

Hip fractures are a severe health problem in patients of advanced age because they can cause a significant decline in mobility and can decrease life expectancy [4]. Osteoporosis has a strong association with systemic metabolic disorders. Arterial calcification processes share common pathways with bone physiology, particularly osteoporosis [10]. Rhee et al. evaluated the relationship between receptor activator of nuclear factor-kappaB ligand (RANKL) gene polymorphism and aortic calcification [11]. Akune et al. showed that insulin receptor substrate signaling caused a potent bone anabolic action by insulin and insulin-like growth factor-I [12]. Furthermore, osteopontin, a phosphoglycoprotein relevant to the immune response, was upregulated in cases with low bone mineral density [13]. Considering these findings, intervention for osteoporosis is crucial to prevent fractures.

NLR and MLR have been studied and were found valuable for predicting the outcomes or prognosis of oncological diseases. Cook et al. found that an NLR value ≥9.3 on the first POD following colorectal surgery was associated with increased mortality [14]. Yasui et al. reported that postoperative, but not preoperative, inflammation-based prognostic markers more accurately predicted prognosis in patients with colorectal cancer [15]. In orthopedics, an increased NLR after hip fracture operations was a risk factor for postoperative mortality [16, 17]. MLR has a higher diagnostic value for osteoporosis [18]. We postulated that patients with YAM <70% had higher inflammatory biomarker concentrations owing to the greater stress they sustained from surgery compared with patients with YAM >70%. As a result, our outcomes showed that patients with YAM >70% had lower NLR and MLR values on both POD 1 and POD 7. There was no correlation between the laboratory data and YAM in patients with YAM <70%. The results of the ROC curve analysis demonstrated that both NLR and MLR on both POD 1 and POD 7 showed moderate accuracy. In particular, the specificity of MLR on POD 1 was quite high. These results suggested that YAM >70% was an appropriate intervention threshold.

Several elements may be relevant to postoperative recovery after hip fracture surgery. In the current study, low BMI was associated with BMD <70%. Reginster et al. reviewed the link between osteoporosis and sarcopenia [19], and age-related loss of skeletal muscle was associated with cardiovascular mortality in another study [20]. Treatment to increase muscle volume in addition to BMD may help decrease surgical invasiveness and patients' subsequent healthy life expectancy.

## 5. Conclusions

The diagnosis of osteoporosis is based on bone mineral density measurements expressed as a percentage of the young adult mean (YAM) in Japan. Osteoporosis is defined as YAM <70%. Because osteoporosis has a strong association with systemic metabolic disorders, we postulated that patients with YAM <70% had higher inflammatory biomarker concentrations owing to the higher systemic stress compared with YAM >70%. Patients with YAM >70% had lower NLR and MLR values on POD 1 and POD 7 after hip surgery. YAM cutoff of <70% was an appropriate intervention threshold regarding postoperative recovery after hip fracture surgery.

## Figures and Tables

**Table 1:** Clinical parameters.

Variable	YAM ≥ 70% (*n* = 17)	YAM < 70% (*n* = 77)	*P* value
Gender male/female	2/15	16/61	0.51
Age (years)	84 (73–89.5)	88 (81.5–92)	0.18
BMI	21.9 (20–23.6)	19.5 (17.5–21.9)	**0.006**
*ASA-PS*			0.45
2	8	44	
3	9	33	
*Type of fracture*			0.94
Neck	9	40	
Trochanteric	8	37	
*Surgery*			0.58
BHA	5	9	
ORIF	12	68	
DM±	6/22	2/22	0.12
HT±	10/7	49/28	0.71

ASA-PS: American Society of Anesthesiologists physical status, BHA: bipolar hip arthroplasty, BMI: body mass index, DM: diabetes mellitus, HT: hypertension, *n*: number of patients, ORIF: open reduction and internal fixation, YAM: young adult mean.

## Data Availability

The data that support the findings of this study are available from the corresponding author.
